# Oligo-Carrageenans Enhance Growth and Contents of Cellulose, Essential Oils and Polyphenolic Compounds in *Eucalyptus globulus* Trees 

**DOI:** 10.3390/molecules18088740

**Published:** 2013-07-24

**Authors:** Alberto González, Rodrigo A. Contreras, Alejandra Moenne

**Affiliations:** Marine Biotechnology Laboratory, Faculty of Chemistry and Biology, University of Santiago of Chile, Casilla 40 Correo 33, Santiago 9170022, Chile; E-Mails: alberto.ngf@gmail.com (A.G.); rodrigo.contrerasar@usach.cl (R.C.)

**Keywords:** cellulose, essential oils, *Eucalyptus globulus*, growth, oligo-carrageenans, polyphenolic compounds, photosynthesis

## Abstract

*Eucalyptus globulus* (Myrtaceae) originated in Australia and has been introduced in countries with temperate weather in order to obtain wood for cellulose extraction and building purposes. In this work, we analyzed the potential stimulation of growth in height and trunk diameter as well as the content of holo-cellulose, α-cellulose (long cellulose chains), essential oils and polyphenolic compounds (PPCs) in *E. globulus* trees treated with oligo-carrageenans (OCs) kappa, lambda and iota, at 1 mg mL^−1^, once a week, four times in total and then cultivated for three additional years without further treatment. *Eucalyptus* treated with OCs kappa, lambda and iota showed an increase in height, mainly with OCs kappa and iota by 58% and 47%, respectively, and in trunk diameter by 44% and 40%, respectively. In addition, OCs induced an increase in the contents of holo-cellulose and α-cellulose, mainly OCs kappa and iota which increased holo-cellulose by 8% and 5%, respectively, and α-cellulose by 16 and 13%, respectively. Moreover, OCs increased the content of essential oils, mainly OCs kappa and iota by 67% and 39%, respectively. Furthermore, OCs decreased the concentration of total phenolic compounds but differentially changed the concentration of several PPCs such as genistein, rutin, ellagic acid, morin, luteolin and quercetin with potential antimicrobial activities. Thus, marine algae OCs kappa, lambda and iota stimulate growth of *E. globulus* trees by enhancing height and trunk diameter as well as the content of α-cellulose, total essential oils, and some PPCs with potential antimicrobial activities.

## 1. Introduction

*Eucalyptus* species belong to the family Myrtaceae originated in Australia where more than 600 species have been identified. *Eucalyptus globulus* trees display rapid growth and they have been introduced in countries with temperate weather as a source for cellulose extraction, building material and to obtain essential oils from leaves for pharmaceutical uses [1].

Wood in adult trees is constituted by around 50% of cellulose, 20% hemi-cellulose and 30% lignin [2]. Wood of *Eucalyptus* trees is processed in order to remove lignin and hemicellulose and recover α-cellulose (long cellulose chains) to produce paper and cardboard. Cellulose is a polymer constituted by 50 to 14,000 units of glucose linked by β-1,4-glycosidic bonds and cellulose fibers are arranged as highly ordered parallel microfibrils [3]. Cellulose is synthesized at the plasma membrane by the enzyme cellulose synthase that uses UDP-glucose as substrate which is provided by the enzyme sucrose synthase [4]. 

Leaves of *E. globulus* trees contain essential oils which are mainly terpenes and terpenoids showing antioxidant, anti-inflammatory and antimicrobial properties [5,6]. Essential oils of *E. globulus* are constituted mainly by the monoterpenes 1,8-cineole (eucalyptol, 60%), α-pinene (30%) and D-limonene (5%) and the sesquiterpene aromadendrene [7]. Eucalyptol has antifungal, antibacterial and antiviral activity *in vitro* [8,9,10,11]. In addition, eucalyptol and aromadendrene have synergistic effects in regard to antioxidant and antimicrobial properties [9]. Moreover, it has been determined that *E. globulus* leaves contain several polyphenolic compounds such as ellagic acid, gallic acid, caffeic acid, chlorogenic acid, luteolin, rutin and quercetin in free or conjugated forms which have antioxidant and antimicrobial properties [12,13,14,15]. 

In previous studies, we demonstrated that marine algae oligo-carrageenans (OCs) kappa, lambda and iota stimulate growth as well as defense against several pathogens in tobacco plants [16,17]. OCs kappa, lambda and iota were obtained by acid hydrolysis of pure commercial carrageenans [18]. These OCs are constituted by around 20 units of sulphated galactose linked by alternate β-1,4- and α-1,3- glycosidic bonds with sulphate groups located in positions 2, 4 and 6 of the galactose ring with or without anhydrogalactose units (for a model see [17]). In tobacco plants, optimal results were obtained with OCs at a concentration of 1 mg mL^−1^ and spraying the leaves once a week, four times in total [19]. In tobacco plants treated with OCs, the increase in growth is due, at least in part, to an increase in net photosynthesis, activities of NAD(P)H-synthesizing enzymes involved in basal metabolism and cell division [18]. In addition, OCs induced a long-term and broad-range protection against pathogens such Tobacco Mosaic Virus (TMV), the fungus *Botrytis cinerea* and the bacteria *Pectobacterium carotovorum* in tobacco plants as well as suppression of bacterial, fungal and viral infections [20].

In order to continue the analysis of OCs effects in trees, we treated *E. gobulus* with water (control) or with OCs kappa, lambda and iota at a concentration of 1 mg mL^−1^, once a week, four times in total, and then cultivated them for three additional years without further treatment. We determined height and trunk diameter, net photosynthesis and content of chlorophyll *a* and *b* in control and treated *Eucalyptus* trees. In addition, we analyzed the amount of holo-cellulose (cellulose and hemicellulose) and α-cellulose (long cellulose chains) as well as the level of essential oils (terpenes) and several polyphenolic compounds (PPCs) with potential antimicrobial activities.

## 2. Results and Discussion

### 2.1. Oligo-Carrageenans Increased Growth, Net Photosynthesis and Contents of Chlorophylls in Eucalyptus Trees

**Figure 1:**
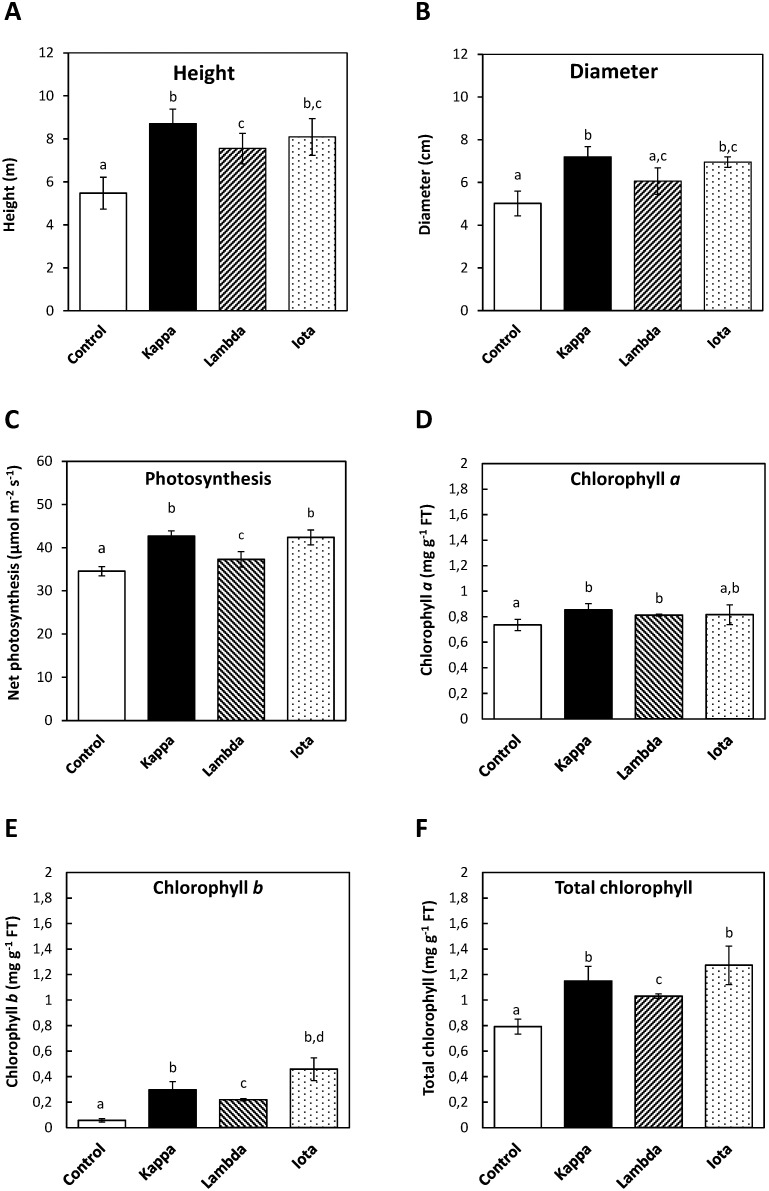
Height (**A**) and trunk diameter (**B**), net photosynthesis (**C**), and contents of chlorophyll *a* (**D**) and *b* (**E**) and total chlorophyll (**F**) in control *Eucalyptus* (control) and in trees treated with oligo-carrageenans (OCs) kappa, lambda and iota.

*E. globulus* trees treated with all three OCs showed an increase in height and trunk diameter compared to controls (Figure 1). The average height of control *Eucalyptus* was 5.5 m whereas that of trees treated with OCs kappa, lambda and iota it was 8.7, 7.6 and 8.1 m, respectively (Figure 1A). Thus, the higher increases in height were observed with OCs kappa and iota and correspond to 58% and 47%, respectively. In addition, mean value of trunk diameter in control *Eucalyptus* was 5 cm and in trees treated with OCs kappa, lambda and iota it was 7.2, 6.1 and 7 cm, respectively (Figure 1B). Thus, the higher increases in trunk diameter were obtained with OCs kappa and iota and correspond to 44% and 40%, respectively. 

Net photosynthesis increased in *Eucalyptus* treated with all three OCs compare to controls. Mean values of net photosynthesis in control *Eucalyptus* was 34.6 µmol m^−2^ s^−1^ whereas in trees treated with OCs kappa, lambda and iota it was 42.7, 37.3 and 42.4 µmol m^−2^ s^−1^, respectively (Figure 1C). Thus, net photosynthesis increased in treated *Eucalyptus*, mainly with OCs kappa and iota, by 23%. 

Levels of chlorophyll *a*, chlorophyll *b* and total chlorophyll increased in *Eucalyptus* treated with all three OCs. Average values of chlorophyll *a*, chlorophyll *b* and total chlorophyll in control *Eucalyptus* trees were 0.74, 0.06 and 0.8 mg g^−1^, respectively, in *Eucalyptus* treated with OC kappa they were 0.85, 0.3 and 1.15 mg g^−1^, respectively, in eucalyptus treated with OC lambda they were 0.81, 0.22 and 1.03 mg g^−1^, respectively, and in *Eucalyptus* treated with OC iota they were 0.81, 0.46 and 1.27 mg g^−1^, respectively (Figure 1D–F). Thus, similar increases in chlorophyll *a* level were observed in *Eucalyptus* treated with OCs kappa, lambda and iota whereas chlorophyll *b* and total chlorophyll showed a higher increase in *Eucalyptus* treated with OC iota.

The increase in height and trunk diameter observed in *Eucalyptus* treated with OCs is due, at least in part to the increase in net photosynthesis which ensures the synthesis of NADPH required by Calvin-Benson cycle enzyme activities allowing carbon (C) assimilation. Considering that photosynthesis increased in treated *Eucalyptus*, it is possible that nitrogen (N) and sulfur (S) assimilations may also be increased since C, N and S metabolism are coordinated light-dependent processes [21,22]. In addition, the increase in total chlorophyll is in accord with the increase in net photosynthesis since light capture and electron transport is previous to C assimilation. Considering that photosynthesis produces NADPH, it is possible to postulate that OCs may increase NADPH content changing the intracellular redox state to a more reducing condition which, in turn, may favor cell division and growth, but these assumptions remained to be determined. Furthermore the increase in photosynthesis observed in *Eucalyptus* treated with all three OCs after 3 years of treatment indicates that OCs induced a stable change in C assimilation and probably in expression of several genes involved in C, N and S assimilation and other metabolic pathways suggesting that an epigenetic change may have occur, but this assumption remained to be determined.

### 2.2. Oligo-Carrageenans Increased Holo-Cellulose and α-Cellulose Contents in Eucalyptus Trees

**Figure 2:**
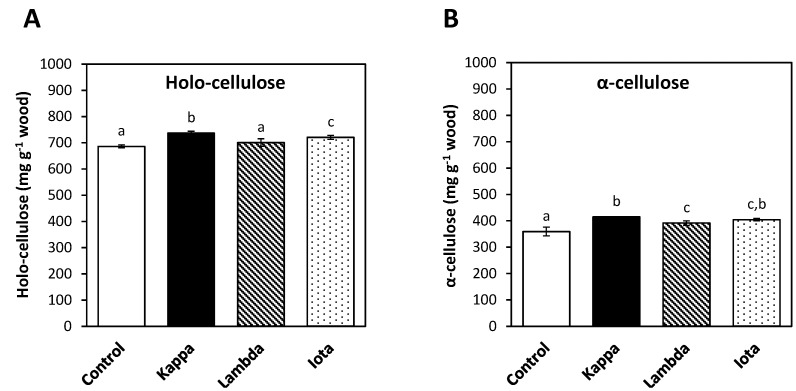
Contents of holo-cellulose (**A**) and α-cellulose (**B**) in branches of control *Eucalyptus* (control) and in trees treated with oligo-carrageenans (OCs) kappa, lambda and iota.

Holocellulose and α-cellulose contents increased in in branches of *E. globulus* treated with all three OCs compare to controls (Figure 2). The content of holo-cellulose in control *Eucalyptus* was 685 mg g^−1^ of wood whereas in trees treated with OCs kappa, lambda and iota it was 737, 700 and 720 mg g^−1^ of wood, respectively (Figure 2A). Thus, the content of holo-cellulose in *Eucalyptus* treated with OCs kappa and iota increased in 8 and 5%, respectively. In addition, the content of α-cellulose in control *Eucalyptus* was 359 mg g^−1^ of wood and in trees treated with OCs kappa, lambda and iota it was 415, 391 and 404 mg g^−1^ of wood, respectively (Figure 2B). Thus, the increase in α-cellulose content in *Eucalyptus* treated with OCs kappa and iota was 16 and 13%, respectively. 

Wood of *E. globulus* trees is used to produce α-cellulose which corresponds to long cellulose fibers. It is important to note that *Eucalyptus* treated with OCs showed an increase in holo-cellulose which indicates that there is a decrease in lignin content, as well as an increase in α-cellulose. Thus, OCs induced a double beneficial effect concerning cellulose extraction from *E. globulus* wood corresponding to a decrease in lignin and an increase in α-cellulose contents. It is interesting to note that the increase in cellulose content of 16% correlates with the increase in net photosynthesis of around 20% which is in accord with the fact that cellulose, hemicellulose and lignin are carbon polymers and, thus, their synthesis is completely dependent on C assimilation [2].

### 2.3. Oligo-Carrageenans Increased Content of Essential Oils in Eucalyptus Trees

**Figure 3:**
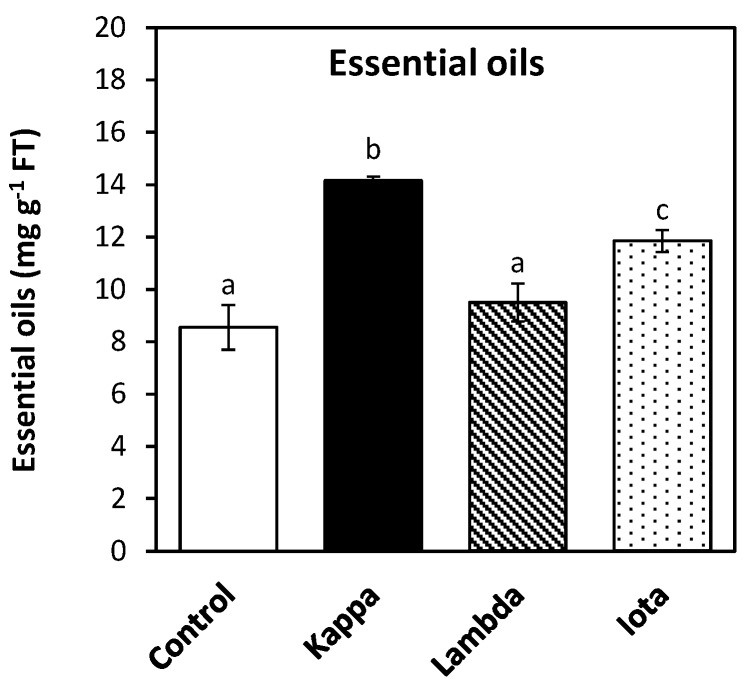
Content of total essential oils in leaves of control *Eucalyptus* (control) and in trees treated with oligo-carrageenans (OCs) kappa, lambda and iota. The amount of essential oils is expressed as milligram per gram of fresh tissue (FT).

The content of essential oils increased in leaves of *E. globulus* treated with all three OCs compare to controls (Figure 3). The amount of essential oils in control *Eucalyptus* was 8.5 mg g^−1^ of fresh tissue (FT) and in trees treated with OCs kappa, lambda and iota it was 14.2, 9.5 and 11.8 mg g^−1^ of FT, respectively (Figure 3). The higher increases were observed in *Eucalyptus* treated with OCs kappa and iota they were 67 and 39%, respectively. Thus, the content of essential oils increased in *E. globulus* trees treated with all three OCs more so with kappa form. It has been shown that essential oils of *E. globulus* have antifungal [9], antibacterial [8] and antiviral [10] activities *in vitro*. Thus, the increased content of essential oils in *Eucalyptus* trees treated with OCs may induce protection against fungal, bacterial and/or viral infections, but this assumption remained be to be determined. 

### 2.4. Oligo-Carrageenans Changed the Level of Polyphenolic Compounds in Eucalyptus Trees

**Figure 4:**
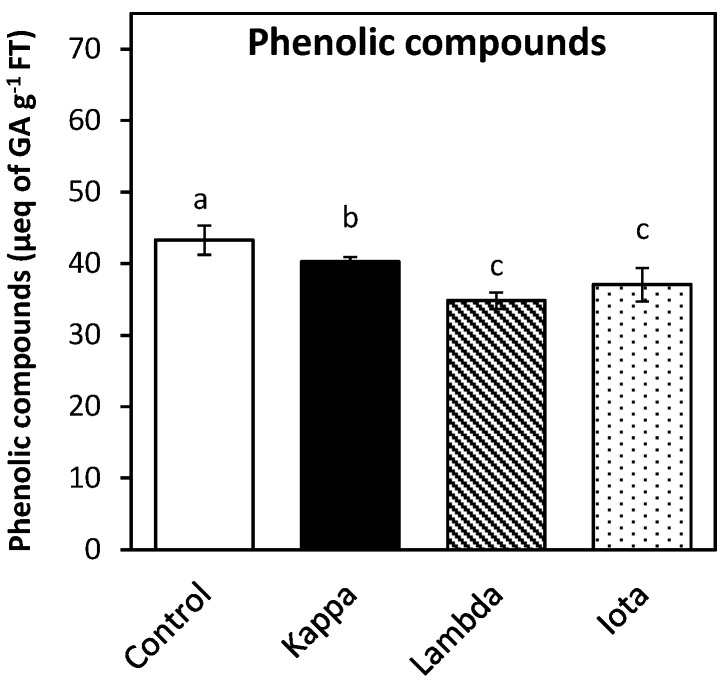
Total phenolic compounds in leaves of control *Eucalyptus* (control) and in trees treated with OCs kappa, lambda and iota. The amount of total phenolic compounds is expressed in microequivalents of gallic acid per gram of fresh tissue (FT).

The level of total phenolic compounds decreased in leaves of *E. globulus* treated with OCs kappa, lambda and iota (Figure 4). The content of total phenolic compounds in control *Eucalyptus* was 43 microequivalents of gallic acid g^−1^ of FT and in trees treated with OCs kappa, lambda and iota it was 40, 35 and 37 micromoles of gallic acid g^−1^ of FT, respectively. The decrease in total phenolic compounds in *Eucalyptus* treated with lambda and iota OCs corresponds to 18% and 14%, respectively (Figure 4). However, the level of several methanol-extracted polyphenolic compounds (PPCs) increased in leaves of *Eucalyptus* trees treated with OCs whereas others decreased compare to controls. In general, the level of 29 PPCs changed in *Eucalyptus* treated with OCs kappa, lambda and iota of which 8–15 increased depending on the OC used and 13–20 decreased but most of them could not be identified (data not shown). Seven methanol-extracted PPCs were identified corresponding to ellagic acid, quercetin, morin, rutin, luteolin, genistein and sinapic acid (Table 1). Interestingly, some PPCs such as caffeic acid, ferulic acid, chlorogenic acid, vainillic acid, galic acid, escopoletin, esculetin, reverastrol, apigenin, naringenin and kaempferol were not detected. The levels of rutin and genistein increased in *Eucalyptus* treated with OC kappa by 200 and 300%, respectively. The levels of ellagic acid, morin and genistein increased in *Eucalyptus* treated with OC lambda by 50, 18 and 550%, respectively. The level of rutin and genistein increased in *Eucalyptus* treated with OC iota by 100 and 450%, respectively. Thus, a phenomenal increase in genistein was observed in *Eucalyptus* treated with OCs kappa, lambda and iota. In contrast, the level of quercetin, morin and luteolin decreased in *Eucalyptus* treated with OC kappa, the level of quercetin, rutin and luteolin decreased in trees treated with OC lambda and the level of morin and luteolin decreased in *Eucalyptus* treated with OC iota. Thus, OCs kappa, lambda and iota differentially changed the levels of the six identified PPCs in *Eucalyptus* trees. *E. globulus* treated with OCs showed a higher content of some PPCs such as genistein, rutin, morin, and ellagic acid which are secondary metabolites having antimicrobial properties [23,24,25]. The isoflavone genistein and some of its isoflavan derivatives showed antifungal activity against plant fungal pathogens *in vitro* [24] and antibacterial effect *in vitro* against some human pathogens by inhibiting DNA, RNA and protein synthesis [23]. Moreover, the flavonoid rutin has antifungal activity against a plant fungal pathogen and it enhanced antibacterial activity of the flavonoids morin and quercetin *in vitro* [25]. Furthermore, glycoside derivatives of the tannin ellagic acid displayed antifungal activity against a plant pathogen *in vitro* [26] as well as antimicrobial activity against several human oral pathogens [26]. Thus, the enhanced level of some PPCs having antimicrobial properties may induce protection against fungal, bacterial and/or viral pathogens in *Eucalyptus* trees. In addition, the increase in essential oils combined with the increase of some PPCs levels may further enhance protection against pathogens in *Eucalyptus* trees, but this assumption remained to be determined. 

**Table 1:** Content of polyphenolic compounds (PPCs) in leaves of control *Eucalyptus* (control) and in trees treated with oligo-carrageenans (OCs) kappa lambda and iota.

	PPC contents (mg g^−1^ of fresh tissue)			
	Control	Kappa	Lambda	Iota
Ellagic acid	0.6	0.6	0.9	0.6
Quercetin	2.5	1.6	1.5	2.5
Morin	1.7	0.1	2.0	1.4
Rutin	0.1	0.3	0.0	0.5
Luteolin	2.6	1.7	1.9	2.4
Genistein	0.2	0.8	1.3	1.4
Sinapic acid	1.7	1.2	1.4	1.7

It is important to mention that all three OCs induced differential changes in the accumulation of some PPCs indicating that these oligosaccharides may activate different signal transduction pathways. This is in accord with results obtained in tobacco plants treated with OCs kappa, lambda and iota where a differential accumulation of PPCs was observed [20]. Finally, it is important to point out that various PPCs such as caffeic acid, ferulic acic, chlorogenic acid, vanillic acid, gallic acid, escopoletin, esculetin, kaempferol, naringenin, apigenin and reverastrol were not detected in control or treated *Eucalyptus* trees which contrast with the detection of gallic acid, caffeic acid and chlorogenic acid in *E. globulus* by others [12]. The absence of some PPCs in control and treated *Eucalyptus* trees may be due to the solvent used for extraction as it has been described elsewhere [27].

## 3. Experimental

### 3.1. Preparation of Oligo-Carrageenans

Twenty grams of pure (free of proteins and secondary metabolites) commercial kappa2, lambda and iota carrageenans (Gelymar S.A., Santiago, Chile) were solubilized in 2 L of water at 60 °C. Concentrated HCl (36.2 N) was added to reach a final concentration of 0.1 N, the solution was incubated for 45 min at 60 °C and then NaOH 1 M was added to obtain pH 7. A sample of 10 µL of each depolymerized carrageenan (oligo-carrageenans, OCs) was analyzed by electrophoresis in an agarose gel (1.5% w/v) using 100 V for 1 h and dextran sulphate of 8 and 10 kDa as standards (Sigma, St Louis, MO, USA). The gel was stained with 15% w/v Alcian blue dye in 30% v/v acetic acid/water for 1h at room temperature and washed with 50% v/v acetic acid/water for 1 h. OCs kappa, lambda and iota were visualized as a relative discrete band of around 10 kDa.

### 3.2. Plant Culture, Treatment and Measurement of Height and Trunk Diameter

*E. globulus* trees were cultivated outdoors in plastic bags containing compost for one year, from spring 2008 until spring 2009, and then transferred to the field and cultivated for two additional years. *E. globulus* trees having an initial height of 25 cm were sprayed in the upper and lower part of the leaves with 2 mL of water per plant (control group, *n =* 7) or with 2 mL of an aqueous solution of OCs kappa, lambda or iota (each group *n =* 7) at a concentration of 1 mg mL^−1^, once a week, four times in total and cultivated without any additional treatment for 3 years. The height of trees was determined using a measuring tape and the trunk diameter using a caliper.

### 3.3. Determination of Net Photosynthesis

Net photosynthesis was measured in control and treated *Eucalyptus* trees (*n =* 5 for each group) in five leaves located in the middle part of the tree using a portable infrared gas analyzer Ciras-1 (PP Systems, Hitchin, UK), with a leaf cuvette of 12.5 cm^2^ using a red/white LED light source, a photon irradiance of 1,000 µmol quanta m^−2^ s^−1^ photosynthetic active radiation (PAR), a CO_2_ concentration of 300 ppm and a relative humidity of 70% at 24 °C for 1 min.

### 3.4. Determination of Chlorophyll a and b Contents

Leaves (6 g of fresh tissue) of control and treated *Eucalyptus* trees (*n =* 5 for each group) were freezed in liquid nitrogen and homogenized in a mortar with a pestle. Twelve mL of hexane-acetone (3:1) were added and the mixture was incubated overnight at room temperature. The mixture was filtrated on Miracloth paper (Calbiochem, Darmstadt, Gemany) and the absorbance of chlorophyll *a* and *b* was detected at 663 and 646 nm, respectively, using Hewlett-Packard/Agilent spectrophotometer model 8453 (Santa Clara, CA, USA). The levels of chlorophylls *a* and *b* were calculated as described in [28] using the formula:

Chlorophyll *a* (μg mL^−1^) = 12.5 A_663_ – 2.79 A_646_

Chlorophyll *b* (μg mL^−1^) = 20.5 A_646_ – 5.1 A_663_

### 3.5. Determination of Holo-Cellulose and α-Cellulose Contents

The contents of holo-cellulose and α-cellulose were determined as described in [29]. Wood of branches (50 g) of control and treated *Eucalyptus* trees (*n =* 3 for each group) were homogenized to obtain saw dust using a wood mill. *Eucalyptus* saw dust was sieved using steel sieves with mesh size of 422 and 251 μm (Dual Manufacturing, Chicago, IL, USA). Size-fractionated saw dust (3 g) was added to 60 mL of distilled water and 6 mL of acetic acid and then 15 mL of 10% (w/v) sodium chlorite were added and the mixture was incubated for 30 min at 90 °C. Acetic acid and sodium chlorite were added three more times and the mixture was incubated in similar conditions. Reaction was stopped by incubation on ice and the mixture was filtered using a porcelain Gooch filter with porosity grade of 50–90 μm (Schott AG, Mainz, Germany). Holo-cellulose was washed with 150 mL of water for three times, dried in an oven at 105 °C for 16 h and weighted in a precision balance. 

Holo-cellulose (1 g) was incubated in 80 mL of 17.5% (w/v) NaOH for 30 min at room temperature, 80 mL of distilled water were added and the mixture was incubated for 30 min at room temperature. The mixture was filtered using a porcelain Gooch filter with porosity grade of 50–90 μm, α-cellulose was washed with 600 mL of distilled water three times, incubated in 10 mL of 1 M acetic acid for 5 min, washed three times with 1 L of distilled water, dried in an oven at 105 °C for 16 h and weighted using a precision balance. 

### 3.6. Determination of Essential Oils Content

Leaves (50 g of fresh tissue) of control and treated *Eucalyptus* trees (*n =* 3 for each group) were homogenized in a food mill and added to 500 mL of distilled water. Essential oils were distilled using a Clevenger apparatus for 45 min. Essential oils which have a lower density than water were recovered with a micropipette and weighted using a precision balance. 

### 3.7. Determination of Total Phenolic Compounds

Leaves (0.2 g of fresh tissue) of control and treated *Eucalyptus* trees (*n =* 3 for each group) were homogenized in 1 mL of ethanol 85% (v/v) using a plastic tube and pestle. The homogenate was centrifuged at 7,500 g for 10 min using an Eppendorf microcentrifuge and the supernatant was recovered. A sample of 100 µL was mixed with 50 µL of Folin-Ciocalteau reagent (Merck, Darmstadt, Germany) and 700 μL of water and the solution was incubated for 1 h at room temperature. To stop the reaction 150 µL of 7% sodium carbonate were added and the absorbance was determined at 765 nm. The level of total phenolic compounds was expressed as microequivalents of gallic acid (Sigma) using a standard curve prepared with 0 to 50 micromoles of gallic acid in a final volume of 1 mL.

### 3.8. Analysis of Methanol-Soluble Polyphenolic Compounds

Leaves (5 g of fresh weight) of control and treated *Eucalyptus* trees (*n =* 3 for each group) were freezed in liquid nitrogen and pulverized in a mortar with a pestle. Fifty milliliters of 100% methanol were added, the mixture was incubated in darkness for 24 h at room temperature, centrifuged at 7,400 g for 15 min and the supernatant was recovered. An aliquot of 20 µL of methanol-soluble PPCs was analyzed by HPLC using an Agilent equipment model 1110, a reversed phase C-18 column (15 cm length, 4.6 mm inner diameter and 5 µm particle size) coupled to a photodiode array detector. PPCs were eluted with a gradient of 1% (v/v) phosphoric acid (A) and acetonitrile (B) constituted by steps of 10% of B for 0 to 5 min, 10 to 25% of B for 5 to 8 min, 25 to 35% of B for 8 to 15 min, 35% of B for 15 to 17 min and 35 to 10% of B for 17 to 20 min, with a flow rate of 1 mL min^−1^ at 25° C. PPCs were detected and identified at 254, 280, 314 and 340 nm using absorption spectra of pure commercial standards (Sigma) corresponding to caffeic acid, ferulic acic, chlorogenic acid, vanillic acid, gallic acid, sinapic acid, escopoletin, esculetin, kaempferol, naringenin, apigenin, reverastrol, ellagic acid, quercetin, morin, rutin, luteolin and genistein and were quantified using a calibration curve prepared with pure standards at concentrations ranging from 0 to 1 mg mL^−1^. 

### 3.9. Statistical Analysis

Significant differences were determined by two-way analysis of variance (ANOVA) followed by Tukey’s multiple comparison tests (*T*). Mean values of height and trunk diameter were obtained from seven *Eucalyptus* trees, photosynthesis was obtained from five trees and holo-cellulose, essential oils and PPC contents were determined using three independent samples. Differences between mean values were considered to be significant at a probability of 5% (*p* < 0.05) [30].

## 4. Conclusions

In summary, marine algae OCs kappa, lambda and iota enhanced height and trunk diameter in *E. globulus* trees which is determined, at least in part, by the increase in net photosynthesis. In addition, OCs induced an increase of holo-cellulose and α-cellulose, essential oils and several PPCs with potential antimicrobial activities. Thus, OCs displayed a double beneficial effect by enhancing growth and cellulose content in *Eucalyptus* trees which may be useful for commercial purposes regarding cellulose extraction from *Eucalyptus* wood.
